# Integrating Crop Growth Models with Whole Genome Prediction through Approximate Bayesian Computation

**DOI:** 10.1371/journal.pone.0130855

**Published:** 2015-06-29

**Authors:** Frank Technow, Carlos D. Messina, L. Radu Totir, Mark Cooper

**Affiliations:** 1 Breeding Technologies, DuPont Pioneer, Johnston, IA, USA; 2 Trait Characterization & Development, DuPont Pioneer, Johnston, IA, USA; University of Nottingham, UNITED KINGDOM

## Abstract

Genomic selection, enabled by whole genome prediction (WGP) methods, is revolutionizing plant breeding. Existing WGP methods have been shown to deliver accurate predictions in the most common settings, such as prediction of across environment performance for traits with additive gene effects. However, prediction of traits with non-additive gene effects and prediction of genotype by environment interaction (G×E), continues to be challenging. Previous attempts to increase prediction accuracy for these particularly difficult tasks employed prediction methods that are purely statistical in nature. Augmenting the statistical methods with biological knowledge has been largely overlooked thus far. Crop growth models (CGMs) attempt to represent the impact of functional relationships between plant physiology and the environment in the formation of yield and similar output traits of interest. Thus, they can explain the impact of G×E and certain types of non-additive gene effects on the expressed phenotype. *Approximate Bayesian computation* (ABC), a novel and powerful computational procedure, allows the incorporation of CGMs directly into the estimation of whole genome marker effects in WGP. Here we provide a proof of concept study for this novel approach and demonstrate its use with synthetic data sets. We show that this novel approach can be considerably more accurate than the benchmark WGP method GBLUP in predicting performance in environments represented in the estimation set as well as in previously unobserved environments for traits determined by non-additive gene effects. We conclude that this proof of concept demonstrates that using ABC for incorporating biological knowledge in the form of CGMs into WGP is a very promising and novel approach to improving prediction accuracy for some of the most challenging scenarios in plant breeding and applied genetics.

## Introduction

Genomic selection [1], enabled by whole genome prediction (WGP) methods, is revolutionizing plant breeding [2]. Since its inception, attempts to improve prediction accuracy have focused on: developing improved and specialized statistical models [3–6], increasing the marker density used [7–9], increasing the size and defining optimal designs of estimation sets [10–13] and better understanding the genetic determinants driving prediction accuracy [14, 15].

In-silico phenotypic prediction, enabled by dynamic crop growth models (CGMs), dates back to the late 1960’s [16] and it has constantly evolved through inclusion of scientific advances made in plant physiology, soil science and micrometeorology [16, 17]. CGMs used in plant breeding are structured around concepts of resource capture, utilization efficiency and allocation among plant organs [18–21] and are used to: characterize environments [22, 23], predict consequences of trait variation on yield within a genotype × environment × management context [24], evaluate breeding strategies [25–27], and assess hybrid performance [2].

Early attempts to extend the use of CGMs to enable genetic prediction have focused on developing genetic models for parameters of main process equations within the CGM [21, 28, 29]. Linking quantitative trait locus (QTL) models and CGMs for complex traits motivated adapting CGMs to improve the connectivity between physiology and genetics of the adaptive traits [21, 27, 30]. However, despite a tremendous body of knowledge and experience, CGMs were largely ignored for the purpose of WGP.

There is ample evidence for the importance of epistasis in crops, including for economically important traits such as grain yield in maize [31–33]. Yield and other complex traits are the product of intricate interactions between component traits on lower hierarchical levels [19, 34–37]. If the relationship among the underlying component traits is nonlinear, epistatic effects can occur on the phenotypic level of complex traits even if the gene action is purely additive when characterized at the level of the component traits [33]. This phenomenon was first described for multiplicative relationships among traits by Richey [38] and later quantified by Melchinger et al. [39]. CGMs, which explicitly model these nonlinear relationships among traits, have therefore the potential to open up novel avenues towards accounting for epistatic effects in WGP models by explicit incorporation of biological knowledge.

The target population of environments for plant breeding programs is subject to continuous re-evaluation [2]. To select for performance in specific environments, genotype by environment (G×E) interactions have to be predicted. Genomic prediction of G×E interactions is therefore of great interest for practical applications of breeding theory. Previous attempts incorporated G×E interactions in WGP models through environment specific marker effects [40] or genetic and environmental covariances [41]. Later Jarquín et al. [42] and Heslot et al. [43] developed WGP models that accounted for G×E interactions by means of environmental covariates.

While these previous attempts are promising, they are purely statistical in nature and do not leverage the substantial biological insights into the mechanisms determining performance in specific environments. CGMs are an embodiment of this biological knowledge and might serve as a key component in novel WGP models for predicting G×E interactions. In fact, Heslot et al. [43] recognized this potential for CGMs. However, they employed them only for computing stress covariates from environmental data, which were subsequently used as covariates in purely statistical WGP models.

Given the potential merits of integrating CGMs in WGP, the question arises of how to combine the two in a unified predictive system. The ever increasing computational power of modern computing environments allows for efficient simulation from the most complex of models, such as CGMs [27]. This computational power is leveraged by *approximate Bayesian computation* (ABC) methods, which replace the calculation of a likelihood function with a simulation step, and thereby facilitate analysis when calculation of a likelihood function is impossible or computationally prohibitive. ABC methods were developed in population genetics, where they helped solve otherwise intractable problems [44–47]. However, ABC methods were rapidly adopted in other scientific fields, such as ecology [48], systems biology [49] and hydrology [50]. Recently, Marjoram et al. [51] proposed using ABC methods for incorporating the biological knowledge represented in gene regulatory networks into genome-wide association studies, arguing that this might present a solution to the ‘missing heritability’ problem.

Here we make the case that ABC may hold great promise for enabling novel approaches to WGP as well. Thus, the objective of this study is to provide a proof of concept, based on synthetic data sets, for using ABC as a mechanism for incorporating the substantial biological knowledge embodied in CGMs into a novel WGP approach.

## Materials and Methods

### CGM and environmental data

We used the maize CGM developed by Muchow et al. [52], which models maize grain yield development as a function of plant population (PPOP, plants *m* ^−2^), daily temperature (°*C*) and solar radiation (*MJ* *m* ^−2^) as well as several genotype dependent physiological traits. These traits were total leaf number (TLN), area of largest leaf (AM), solar radiation use efficiency (SRE) and thermal units to physiological maturity (MTU). Details on the calculation of trait values for the genotypes in the synthetic data set are provided later. However, the values used were within typical ranges reported in the literature. The simulated intervals for TLN, AM, SRE and MTU were [6, 23] [52, 53], [700, 800] [52, 54], [1.5,1.7] [55] and [1050, 1250] [56–58], respectively, with average values at the midpoints of the intervals.

**Fig 1:**
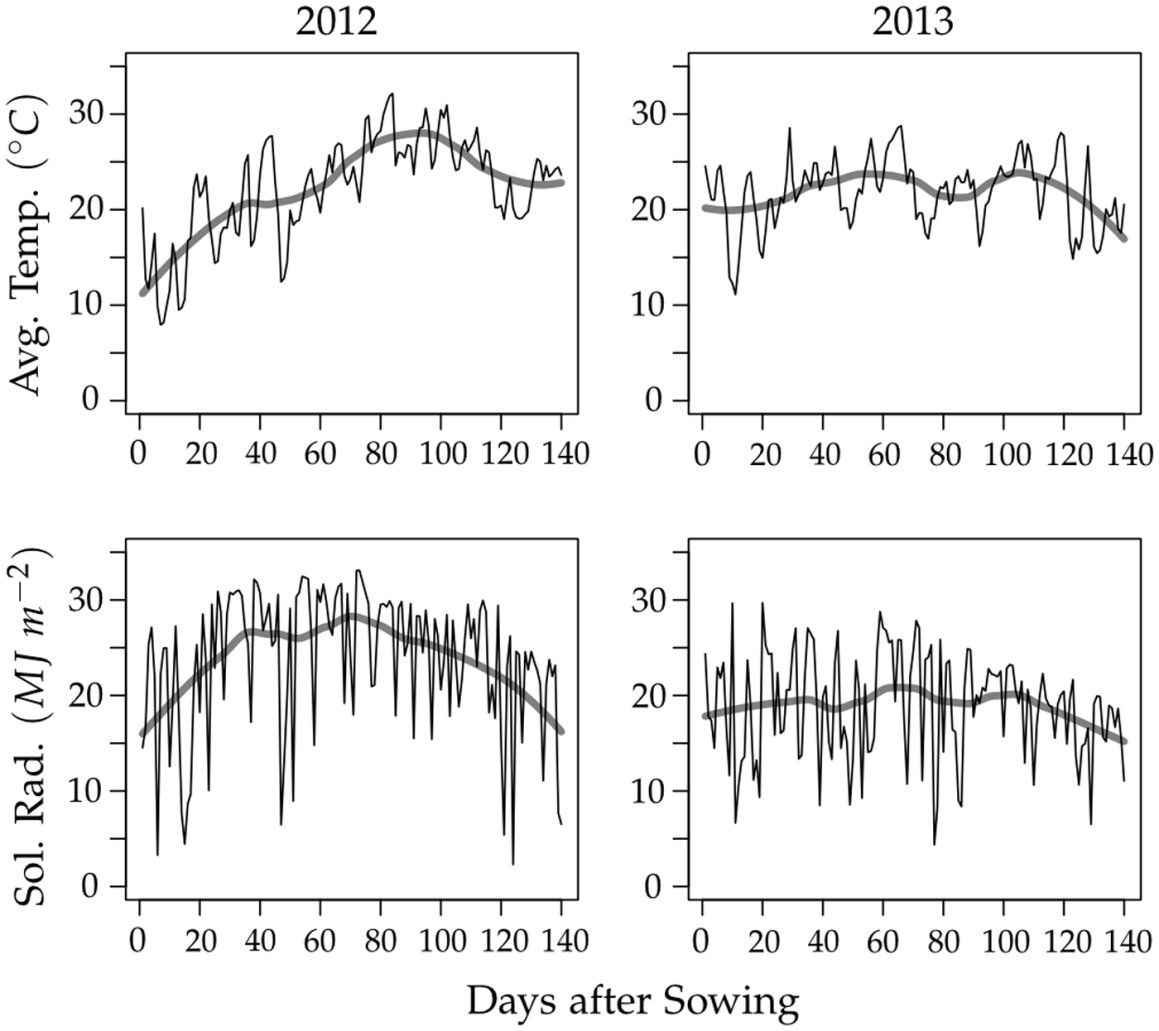
Daily average temperature and solar radiation at Champaign, Illinois in 2012 and 2013. The thick grey line shows a smoothed curve.

**Fig 2:**
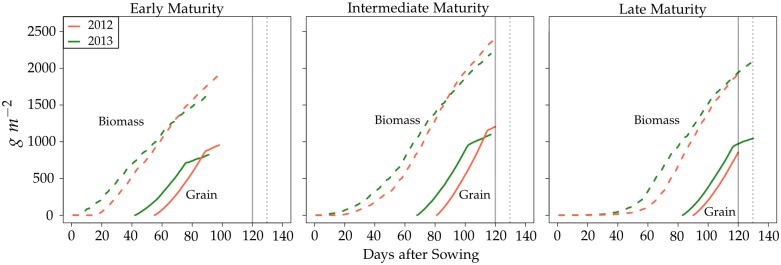
Simulated development of total biomass and grain yield. The early, intermediate and late maturing genotypes had a total leaf number (TLN) of 6, 14.5 and 23, respectively. The values for the other three traits were 750 for AM, 1.6 for SRE and 1150 for MTU and in common for all genotypes. The full and dotted vertical lines indicate the end of the 2012 and 2013 growing season, respectively.

We chose Champaign/Illinois (40.08° N, 88.24° W) as a representative US Corn Belt location. Temperature and solar radiation data were obtained for the years 2012 and 2013 (Data provided by the Water and Atmospheric Resources Monitoring Program, a part of the Illinois State Water Survey (ISWS) located in Champaign and Peoria, Illinois, and on the web at www.sws.uiuc.edu/warm). The sowing date in 2012 was April 15th and in 2013 it was May 15th. We modified the original CGM of Muchow et al. [52] by enforcing a maximum length of the growing season, after which crop growth simulation was terminated, regardless of whether the genotype reached full physiological maturity or not. The length of the growing season in 2012 was 120 days from sowing and in 2013 it was 130 days from sowing. Both durations are within the range typically observed in the US Corn Belt [59]. In 2012 PPOP was 8 plants *m* ^−2^ and in 2013 PPOP was 10 plants *m* ^−2^. The 2012 and 2013 environments therefore differed not only in temperature and solar radiation but also in management practices. The temperature and solar radiation from date of sowing is shown in Fig 1. Typical total biomass and grain yield development curves for early, intermediate and late maturing genotypes in the 2012 and 2013 environments are shown in Fig 2 and corresponding curves for development of total and senescent leaf area in S1 Fig.

The CGM can be viewed as a function *F* of the genotype specific inputs (the physiological traits) and the environment data

$$F(y_{TLN_{i}},y_{SRE_{i}},y_{AM_{i}},y_{MTU_{i}},\Omega_{k})$$ (1)

where *y*_*TLN*_*i*__ etc. are the values of the physiological traits observed for the *i* ^*th*^ genotype and the weather and management data of environment *k* are represented as Ω_*k*_. To simplify notation, we will henceforth use *F*(⋅)_*ik*_ to represent the CGM and its inputs for genotype *i* in environment *k*.

### Approximate Bayesian Computation (ABC)

ABC replaces likelihood computation with a simulation step [44]. An integral component of any ABC algorithm is therefore the simulation model operator $\text{Model}(y_{ik}^{*}|\theta )$ which generates simulated data $y_{ik}^{*}$ given parameters *θ*. In our proof of concept study, the crop growth model *F*(⋅)_*ik*_ represents the deterministic component of $\text{Model}(y_{ik}^{*}|\theta )$, to which a Gaussian noise variable distributed as $𝓝(0,\sigma_{e}^{2})$ is added as a stochastic component. If $\text{Model}(y_{ik}^{*}|\theta )$ is fully deterministic, the distribution sampled with the ABC algorithm will not converge to the true posterior distribution when the tolerance for the distance between the simulated and observed data goes to zero [50].

The weather and management data Ω_*k*_ was assumed to be known, the physiological traits, however, were unknown and treated as latent or hidden variables, that were modeled as linear functions of the trait specific marker effects

$$\begin{array}{cc} y_{TLN_{i}} & =\mu_{TLN}+z_{i}u_{TLN}\\ y_{AM_{i}} & =\mu_{AM}+z_{i}u_{AM}\\ y_{SRE_{i}} & =\mu_{SRE}+z_{i}u_{SRE}\\ y_{MTU_{i}} & =\mu_{MTU}+z_{i}u_{MTU}, \end{array}$$ (2)

where **z**_*i*_ is the genotype vector of the observed biallelic single nucleotide polymorphism (SNP) markers of genotype *i*, *μ*_*TLN*_ etc. denote the intercepts and **u**_*TLN*_ etc. the marker effects. For brevity, we will use *θ* to denote the joint parameter vector [*μ*_*TLN*_,…, *μ*_*MTU*_,**u**_*TLN*_,…,**u**_*MTU*_].

We used independent Normal distribution priors for all components of *θ*. The prior for *μ*_*TLN*_ was $𝓝(m_{TLN},\sigma_{\mu_{TLN}}^{2})$. To simulate imperfect prior information, we drew the prior mean *m*_*TLN*_ from a Uniform distribution over the interval $\left[0.8\cdot \bar{TLN},1.2\cdot \bar{TLN}\right]$, where $\bar{TLN}$ is the observed population mean of TLN. The average difference between *m*_*TLN*_ and $\bar{TLN}$ then is 10% of the latter value. The prior variance $\sigma_{\mu_{TLN}}^{2}$, which represents the prior uncertainty, was equal to 2.25^2^. The prior means of AM, SRE and MTU were obtained accordingly and the prior variances $\sigma_{\mu_{AM}}^{2}$, $\sigma_{\mu_{SRE}}^{2}$ and $\sigma_{\mu_{MTU}}^{2}$ were 150^2^, 0.3^2^ and 225^2^, respectively.

The prior for the marker effects **u**_*TLN*_ was $𝓝(0,\sigma_{\mu_{TLN}}^{2})$, which corresponds to the *BayesC* prior [60]. In BayesC, the prior variance of marker effects $\sigma_{\mu_{TLN}}^{2}$, which introduces shrinkage, is the same across markers. For simplicity, we set this variance to a constant value and did not attempt to estimate it. Also in this case we simulated imperfect information by drawing the value of $\sigma_{\mu_{TLN}}^{2}$ from a Uniform distribution over the interval [0.8 ⋅ *var*(*TLN*)/*M*,1.2 ⋅ *var*(*TLN*)/*M*], where *M* is the number of markers and *var*(*TLN*) the observed population variance of TLN. The prior variances of marker effects of the other traits were obtained accordingly.

The value of $\sigma_{e}^{2}$, the variance of the Gaussian noise variable that is part of the model operator $\text{Model}(y_{ik}^{*}|\theta )$, was drawn from a Uniform distribution over the interval [0.8 ⋅ *v*_*e*_,1.2 ⋅ *v*_*e*_], where *v*_*e*_ is the residual variance component of the phenotypic grain yield values used to fit the model.

Algorithm 1 in Table 1 shows pseudocode for the ABC rejection sampling algorithm we used. As distance measure between the simulated and observed data we used the Euclidean distance. The tolerance level *ϵ* for the distance between the simulated and observed data was tuned in a preliminary run of the algorithm to result in an acceptance rate of approximately 1 ⋅ 10^−6^. The number of posterior samples drawn was 100. We will refer to this ABC based WGP method that incorporates the CGM as *CGM-WGP*. The CGM-WGP algorithm was implemented as a C routine integrated with the R software environment [61].

**Table 1:** Pseudocode of ABC rejection sampling algorithm.

**while** *x* <= no. posterior samples **do**
**while** *d* > *ϵ* **do**
draw candidate θ* from prior(*θ*)
**for All** *i* = 1, 2, …, *N* **do**
generate simulated data $y_{ik}^{*}$ from $\text{Model}(y_{ik}^{*}|\theta^{*})$
**end for**
compute $d=\sqrt{\sum_{i=1}^{N}{\left(y_{ik}-y_{ik}^{*}\right)}^{2}}$
**end while**
accept and store θ*
increment *x*
**end while**

Basic ABC rejection sampling algorithm to sample from the approximate posterior distribution of *θ*.

### Synthetic data set

To test the performance of CGM-WGP, we created a biparental population of 1,550 doubled haploid (DH) inbred lines in silico. The genome consisted of a single chromosome of 1.5 Morgan length. The genotypes of the DH lines were generated by simulating meiosis events with the software package hypred [62] according to the Haldane mapping function. On the chromosome, we equidistantly placed 140 informative SNP markers. A random subset of 40 of these markers were assigned to be QTL with additive effects on either TLN, AM, SRE or MTU. Each physiological trait was controlled by 10 of the 40 QTL, which were later removed from the set of observed markers available for analysis.

The additive substitution effects of the QTL were drawn from a Standard Normal distribution. Raw genetic scores for each physiological trait were computed by summing the QTL effects according to the QTL genotypes of each DH line. These raw scores were subsequently re-scaled linearly to the aforementioned value ranges. Finally, phenotypic grain yield values were created as

$$y_{ik}=F{\left(\cdot \right)}_{ik}+e_{ik},$$ (3)

where *e*_*ik*_ is a Gaussian noise variable with mean zero and variance *v*_*e*_. The value of *v*_*e*_ was chosen such that the within-environment heritability of *y*_*ik*_ was equal to 0.85. We generated 50 synthetic data sets by repeating the whole process. An example synthetic data set is available as supplemental material (S1 Dataset).

### Estimation, prediction and testing procedure

The models were fitted using *N* = 50 randomly chosen DH lines as an estimation set. The remaining 1500 DH lines were used for testing model performance. Separate models were fitted using the 2012 and the 2013 grain yield data of the estimation set lines. The environment from which data for fitting the model was used will be referred to as *estimation environment*. Parameter estimates from each estimation environment were subsequently used to predict performance of the lines in the test set in both environments. Predictions for the same environment as the estimation environment will be referred to as *observed environment predictions* (e.g., predictions for 2012 with models fitted with 2012 data). Predictions for an environment from which no data were used in fitting the model will be referred to as *new environment predictions* (e.g., predictions for 2013 with models fitted with 2012 data).

As a point estimate for predicted grain yield performance in a specific environment, we used the mean of the posterior predictive distribution for the DH line in question. The posterior predictive distribution was obtained by evaluating *F*(⋅)_*ik*_ over the accepted *θ* samples, using the weather and management data Ω_*k*_ pertaining to that environment.

Prediction accuracy was computed as the Pearson correlation between predicted and true performance in the environment for which the prediction was made. The true grain yield performance was obtained by computing *F*(⋅)_*ik*_ with the true values of the physiological traits.

As a performance benchmark we used genomic best linear unbiased prediction (GBLUP [1]). The model is

$$y_{ik}=\beta_{0}+z_{i}u+e_{i}$$ (4)

where *β*_0_ is the intercept, **u** the vector of marker effects and *e*_*i*_ a residual. As before, **z**_*i*_ denotes the marker genotype vector. The GBLUP model was fitted with the R package rrBLUP [63]. GBLUP and BayesC are comparable in their shrinkage behavior because both use a constant variance across markers. For GBLUP, predicted values were computed according to Eq (4) as *β*_0_+**z**_*i*_ **u**. Note that because the conventional GBLUP model does not utilize information about the environment for which predictions are made, observed and new environment predictions are identical.

## Results and Discussion

### Predicting performance in observed environments

**Fig 3:**
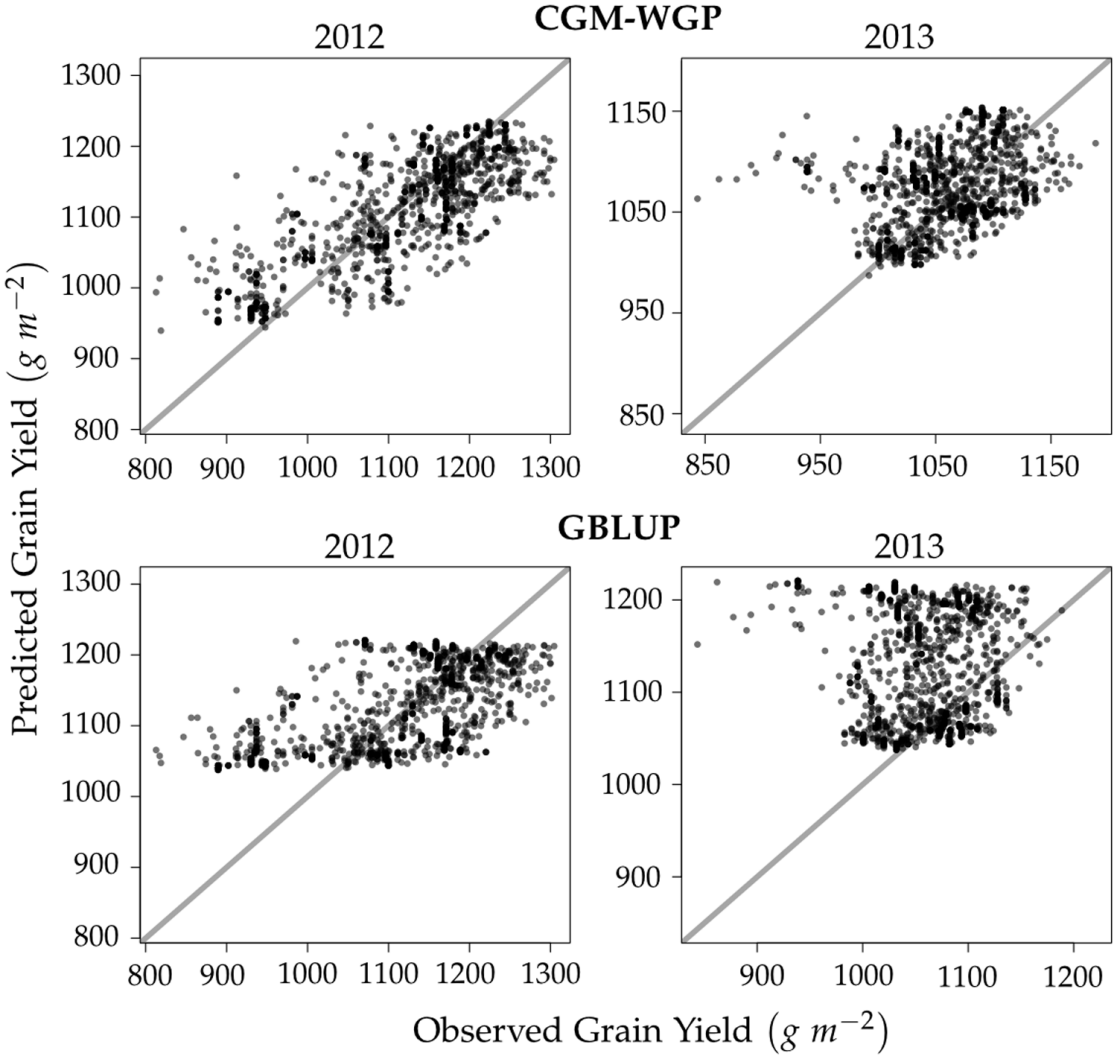
Predicted vs. observed grain yield of 1500 DH lines in testing set for prediction methods CGM-WGP (top row) and GBLUP (bottom row). The estimation environment was 2012. Results shown are from a representative example data set. In this example, the accuracy for observed environment predictions was 0.83 (CGM-WGP) and 0.69 (GBLUP). For new environment predictions it was 0.39 (CGM-WGP) and 0.11 (GBLUP).

**Fig 4:**
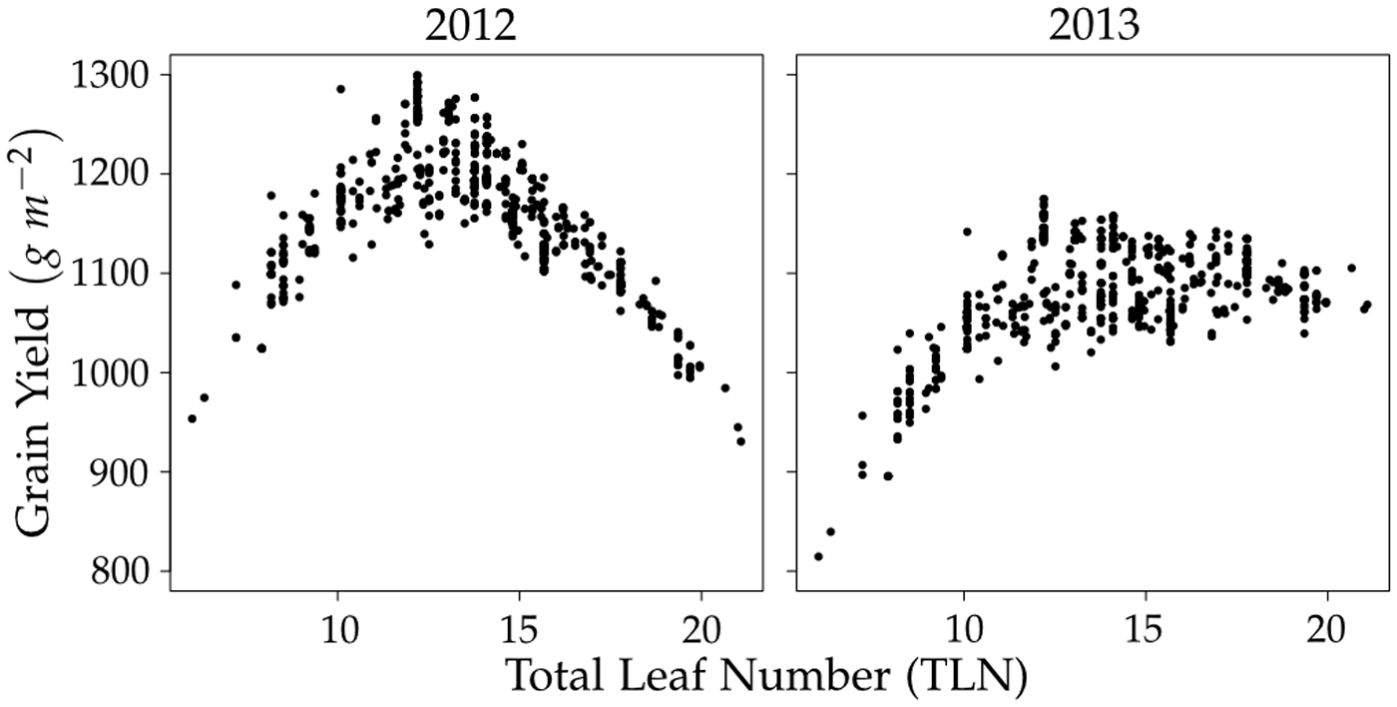
Relationship between total leaf number (TLN) and grain yield. Results shown are from a representative example data set.

The accuracy of observed environment predictions achieved by CGM-WGP was considerably larger than that of the benchmark method GBLUP in both environments (Table 2, Fig 3, S2 Fig). This superiority of CGM-WGP over GBLUP can be explained by the presence of non-additive gene effects which cannot be captured fully by the latter. In the example scenario we studied, the non-additive gene effects on grain yield are a result of nonlinear functional relationships between the physiological traits and grain yield, which was particularly pronounced for TLN (Fig 4).

**Table 2:** Accuracy of grain yield predictions of DH lines in the test set.

Estimation Env.	Prediction Env.	CGM-WGP	GBLUP
2012	2012	0.77	0.54
	2013	0.48	0.10
2013	2012	0.42	0.08
	2013	0.75	0.62

Prediction accuracy for grain yield of DH lines in the test set, averaged over 50 replications.

For any point in time (*t*) during the maize growth cycle, dry matter growth (DM_*g*_) results from the interception of solar radiation (SR) and its conversion into mass with efficiency SRE. Light interception in turn depends on the size of the canopy, which is determined by the leaf area per plant (LAPP) and the plant population (PPOP), and the distribution of light within the canopy, which is modeled using a coefficient of light extinction (*k*). The relationship between DM_*g*_, SR, SRE, LAPP and PPOP is non-linear [52]

$${\text{DM}}_{g_{t}}={\text{SR}}_{t}\times \text{SRE}\times (1-e^{-k\times {\text{LAPP}}_{t}\times \text{PPOP}}).$$ (5)

Because LAPP is determined by TLN and AM [52], DM_*g*_ increases with increasing AM but only up to a point when canopy size maximizes light interception. Because grain yield is a fraction of the integral of DM_*g*_ over the growing season, there is a non-linear relationship between AM and grain yield, which can often be detected as a weak correlation (S3 Fig). From Eq (5) one can see that an increase in SRE is always beneficial for DM_*g*_, which was reflected by the more or less linear relationship between SRE and grain yield (S3 Fig). The longer the length of the period between silking and physiological maturity, the more time the genotype has for grain filling. However, the end of the growing season can forestall exploitation of the longer grain filling periods of high MTU genotypes. Thus, increasing MTU beyond a point determined by the growing season length will have no further effect on grain yield. Such a saturation curve was indeed observed in 2013 (S3 Fig). The relationship between MTU and grain yield tended to be less clear in 2012, where many genotypes did not reach physiological maturity because of the shorter growing season. The relationships between grain yield and the physiological traits AM, SRE and MTU, were generally weak, however, and not obvious in all data sets. We will therefore focus on discussing the relationship between grain yield and TLN, which was very distinct and consistent.

TLN is closely related with the maturity rating of genotypes [52]. The higher it is, the later the onset of the reproductive phase and the later the maturity. Late genotypes have a higher yield potential than earlier genotypes because of a greater leaf area (S1 Fig). However, if the growing season is too short, they cannot realize this yield potential because of their slower development and later onset of the generative phase (Fig 2). Very early genotypes on the other hand, have a low leaf area and do not make use of the full growing season. As a consequence, their realized yield is low, too. The relationship between TLN and grain yield therefore follows an optimum curve (Fig 4). This was particularly pronounced in 2012, which had the shorter growing season and therefore penalized the late maturing genotypes more. The more decidedly nonlinear relationship between grain yield and TLN in 2012 also explains why the difference in prediction accuracy between CGM-WGP and GBLUP was greater in this season than in 2013 (0.23 points in 2012 compared to 0.13 points in 2013, on average).

The scenario we studied is an example of a particular case of epistasis, which might be called *biological epistasis*, that can arise even if the gene effects on the physiological component traits underlying the final trait of interest (grain yield in our case) are purely additive [33]. We accounted for nonlinear functional relationships among traits with the CGM. This enabled us to capture biological epistasis through simple linear models relating marker genotypes to the unobserved underlying physiological traits. Previously developed WGP models attempted to capture epistasis by directly fitting nonlinear marker effects to the final trait of interest [64–66]. While these models showed some promise, they have not been adopted by practitioners on a larger scale. By combining statistics with biological insights captured by CGMs, CGM-WGP takes a fundamentally different approach and presents a potentially powerful alternative to purely statistical WGP models.

### Predicting performance in new environments

New environment prediction accuracy was considerably lower than observed environment prediction accuracy, for both prediction methods (Table 2, Fig 3, S2 Fig). The average prediction accuracy for performance in 2012 when using the 2013 estimation environment was 54% (CGM-WGP) and 15% (GBLUP) of the respective prediction accuracy achieved when using the 2012 estimation environment. The corresponding values for the accuracy of predicting performance in 2013 were 64% (CGM-WGP) and 16% (GBLUP). Thus, CGM-WGP still delivered a decent accuracy for predicting performance in new environments, while GBLUP largely failed in this task. The prediction accuracy of GBLUP was in fact negative, and sometimes strongly so, for close to 50% of the synthetic data sets (S2 Fig). For CGM-WGP negative accuracies were observed in only 14% (2012) and 4% (2013) of the cases.

The genetic rank correlation between true performance in 2012 and 2013 was only 0.54 (averaged over 50 synthetic data sets), which indicated the presence of considerable G×E interactions, including changes in rank (S4 Fig). A genetic correlation of 0.54 between environments is within the range typically observed in plant breeding data sets [67] and crossover interaction between environments is a common phenomenon in plant breeding [34, 68, 69].

The interaction between the environment and TLN again explains the occurrence of G×E to a large degree. In the shorter 2012 season, the late maturing genotypes cannot realize their growth and yield potential and are outperformed by the genotypes with early and intermediate maturity (Figs 2 and 4). In the 10 day longer growing season of 2013, however, the late maturing genotypes can realize their greater yield potential better and outperform the early maturing genotypes and have a similar performance as genotypes with intermediate maturity. This dynamic leads to crossover G×E interactions between the 2012 and 2013 environments.

That new environment prediction under the presence of G×E interaction is considerably less accurate than observed environment prediction was expected and already observed in other studies [11, 70]. It is encouraging that the reduction in accuracy for CGM-WGP was considerably less severe than for the conventional benchmark method GBLUP because this indicates that the former method did succeed in predicting G×E interactions to some degree.

Predicting G×E interactions in new environments for which no yield data are available, requires WGP models that link genetic effects (e.g., marker effects) with information that characterizes the environments. Jarquín et al. [42] accomplished this by fitting statistical interactions between markers and environmental covariates. A similar approach was taken by Heslot et al. [43], who in addition used a CGM to extract stress covariates from a large set of environmental variables. CGM-WGP takes this approach a step further by making the CGM and the environmental data that inform it, an integral part of the estimation procedure.

Nonetheless, while novel prediction methods might succeed in narrowing the gap between new and observed environment prediction, the former should always be expected to be less accurate than the latter. Field testing should therefore be performed in environments of particular importance for a breeding program to achieve the maximum attainable prediction accuracy for these. The same applies for target environments in which G×E interaction effects are expected to be particularly strong. CGMs can help to identify such environments and to inform experimental design and utilization of managed environments [27, 29]. However, the range of the target population of environments of modern plant breeding programs is much too large for yield testing across the whole breadth [2]. Predicting performance in new environments will therefore always be required and novel methods like CGM-WGP are anticipated to be instrumental for enabling and enhancing success in this particularly daunting task.

### Areas of further research and development

#### Alternatives to CGM-WGP

With continual technology improvements for phenotyping traits it is becoming increasingly feasible to assay phenotypic variation for many of the physiological traits underlying the CGM [19]. There would then be no need to treat them as latent, hidden variables as in CGM-WGP. Such improved precision phenotyping capabilities thus open up possible alternatives and extensions to the CGM-WGP methodology introduced here.

One alternative that can be considered is a two-step procedure, in which (1) physiological traits are predicted based on QTL identified in dedicated mapping experiments and (2) the so obtained physiological trait values are used to parametrize CGMs and predict the expected yield performance of novel genotypes in the same or different environments [71–73]. Using WGP instead of QTL mapping in step 1, could further enhance that procedure.

One shortcoming of this approach is that all relevant physiological traits have to be measured for all genotypes in the estimation set. This may prove to be unfeasible in practice, particularly when done on an industrial scale (i.e., for many populations and repeated year after year). The situation is exacerbated when more sophisticated models like APSIM [74], which can model plant-soil interactions related to water and nutrient uptake, are used. The set of relevant physiological traits for these CGMs includes root traits for example [75], which are particularly difficult to measure in a routine, high-throughput fashion [76].

A key novelty of CGM-WGP is that it can accommodate partially or fully unobserved physiological traits by treating them as hidden variables. It could thus facilitate incorporating a CGM in WGP even when phenotyping all relevant physiological traits is not feasible.

However, CGM-WGP and the described two-step approach have a common objective, which is to apply a suitable CGM to capture non-linear relationships among traits and the environment to succeed in the crucial but challenging task of predicting yield in future environments. At this stage it is premature to suggest one approach ahead of these or other possibilities. However, the results of the present study indicate that there are opportunities to improve predictions for quantitative traits influenced by epistasis and G × E interaction, through effective integration of appropriate CGMs into the genetic prediction methodology.

#### More sophisticated CGMs

For this first proof of concept study, we assumed that the CGM used in the estimation process fully represented the systematic component of the data generating process, besides the random noise. This was clearly a ‘best case scenario’. However, decades of crop growth modeling research have provided the know-how necessary to approximate real crop development to a high degree of accuracy [17, 30, 77]. Advanced CGMs such as *APSIM*[74], for example, model functional relationships between various crop parameters and external factors such as water and nutrient availability, soil properties as well as weed, insect and pathogen pressure. Thus, tools are principally available for applying CGM-WGP in more complex scenarios than the one addressed in this study.

With multiple possible CGMs to choose from, model selection becomes an issue. The ABC algorithm underlying CGM-WGP could in principle be used to perform model selection simultaneously with parameter estimation [49, 51, 78]. It could thus provide a statistically formal way of comparing the fit of several CGMs.

#### Stochastic CGMs

There are examples of the use of fully deterministic model operators in ABC [78, 79]. However, with fully deterministic model operators the sampled distribution would not converge to the true posterior when the tolerance level *ϵ* goes to zero [50] and instead reduce to a point mass over those parameter values that can reproduce the data. The CGM we used was fully deterministic. We therefore followed the example of Sadegh and Vrugt [50], who constructed a stochastic model operator by adding a random noise variable, with the same probabilistic properties as assumed for the residual component of the phenotype, to the deterministic functional model. A more elegant and possibly superior solution, however, would be to integrate stochastic processes directly into the CGM. While the vast majority of CGMs are deterministic [16, 17], there are examples of stochastic CGMs [80]. In addition to incorporating inherently stochastic processes of development [81], stochastic CGMs could also serve to account for uncertainty in the parameters of the functional equations comprising the model [82].

#### Advanced ABC algorithms

For this proof of concept study we used the basic ABC rejection sampling algorithm [44, 45]. Considerable methodology related advances have been made, however, over the last decade that have led to algorithms with improved computational efficiency. Of particular interest here are population or sequential Monte Carlo algorithms, which are based on importance sampling [78, 83, 84]. These algorithms can dramatically increase acceptance rates without compromising on the tolerance levels. They achieve this by sampling from a sequence of intermediate proposal distributions of increasing similarity to the target distribution. Unfortunately, importance sampling fails when the number of parameters gets large, because then the importance weights tend to concentrate on very few samples, which leads to an extremely low effective sample size [85]. In the context of sequential Monte Carlo, this is known as particle depletion and was addressed by Peters et al. [84]. We implemented their approach, but were not able to overcome the problem of particle depletion. The number of parameters we estimated was 404 (100 marker effects per physiological trait plus an intercept), which seems well beyond the dimensionality range for importance sampling [85].

Another interesting development is *MCMC-ABC*, which incorporates ABC with the Metropolis-Hastings algorithm [86]. MCMC-ABC should result in high acceptance rates if the sampler moves into parameter regions of high posterior probability. However Metropolis-Hastings sampling too can be inefficient when the parameter space is of high dimension.

The greatest computational advantage of the original ABC rejection algorithm over Monte Carlo based ABC methods is that it generates independent samples and therefore readily lends itself to ‘embarrassingly’ parallel computation [86]. The computation time thus scales linearly to the number of processors available. Using the ABC rejection algorithm therefore allowed us to fully leverage the high performance computing cluster of DuPont Pioneer. In the era of cloud computing [87], high performance computing environments are readily available to practitioners and scientists in both public and private sectors. Generality, scalability to parallel computations, and ease of implementation make the basic rejection sampler a viable alternative to more sophisticated approaches.

#### Using prior information

We used mildly informative prior distributions, the parameters of which were derived from the population means and variances of the physiological traits. In practice, the required prior information must be obtained from extraneous sources, such as past experiments or from the literature [80]. Such information is imperfect and only partially matches the true population parameters of the population in question. We determined the prior parameters from the population itself, but perturbed them considerably to simulate erroneous prior information. Specifically, the average relative discrepancy (bias) between the prior parameter used and the true population parameter was 10%. When we increased the relative discrepancy to 25% (i.e., a maximum discrepancy of 50%), prediction accuracy dropped somewhat (S1 Table). The reduction was only slight for observed environment prediction but more pronounced for new environment prediction. However, CGM-WGP was still considerably more accurate than the benchmark GBLUP. Thus, CGM-WGP seems to be relatively robust to moderate prior miss specification, as long as the value range supported by the prior distribution is not out of scope. In the ideal case of no prior bias, on the other hand, new and observed environment prediction accuracy increased slightly as compared to a bias of 10%.

In the synthetic data sets we generated, the component traits were controlled by QTL with independent effects and thus uncorrelated. In practice, however, the traits might be correlated, because of pleiotropic QTL, for example. In this situation, marker effects are correlated too. We modeled the marker effects as *a priori* independent and note that this does not preclude posterior correlation if CGM-WGP in its current implementation is applied to a scenario with correlated component traits. However, it is possible to model whole genome marker effects as *a priori* correlated. This was explored for the purpose of fitting WGP models with breed specific but correlated marker effects in animal breeding [88]. Modeling a correlation structure could allow information sharing across traits. It could also improve computational efficiency of CGM-WGP, because the prior distribution would be closer to the posterior and thereby result in fewer rejected samples.

Lastly, CGM-WGP can be modified to use ‘BayesB’ [1] or ‘BayesC*π*’ [60] as priors of marker effects, which would allow marker effects to be exactly zero. By allowing the effects of markers to be zero, marker effect estimation and implicit SNP model selection are done simultaneously. This could present an interesting compromise between a continuous WGP approach (BayesC) and a discrete, QTL based approach to prediction.

#### Number of markers

We applied CGM-WGP to a biparental population, which is by far the most common population type in commercial plant breeding programs [89]. Previous WGP studies found that marker density is typically not the most limiting factor in these type of populations and that densities achievable with around 200 genomwide markers suffice for accurate predictions [13, 90–92]. This proof of concept showed that computations for CGM-WGP are feasible for 100 markers. Thus, while more challenging, we expect that computations can be facilitated for the numbers of markers required for biparental populations. However, applying CGM-WGP to data sets with tens of thousands of markers is likely not possible with current ABC algorithms, in particular if more sophisticated CGMs are used that require specification of more physiological traits. Technow and Melchinger [12] showed that using a more realistic but complex WGP model with a lower marker density can result in a higher prediction accuracy than using a less realistic WGP model with a higher marker density. Thus, the greater realism of CGM-WGP might compensate for the fact that it can currently be applied only at low to intermediate marker densities.

In contrast to the complex trait of interest, component physiological traits may be realistically modeled based on a relatively simple genetic architecture, and for such traits, QTL explaining a sizable proportion of genetic variance can be mapped and characterized [71, 93–96]. In fact, such component trait QTL have been successfully used to parametrize CGMs for studying genotype dependent response to environmental conditions [28, 29, 73, 94, 95]. Knowledge about the location and effect of such QTL, or of transgenes [97–99], could be incorporated as an additional source of prior information. Then, instead of estimating marker effects for the whole genome, CGM-WGP could focus on genomic regions of particular importance. This reduces the dimensionality of the parameter space dramatically and enables CGM-WGP to be used in settings that traditionally required high marker densities, such as WGP in diverse germplasm [100].

#### Identifiability

It is possible that the CGM generates the same yield for two or more sets of component trait values. There are often several possible biological strategies with equivalent outcome, so this does not necessarily indicate model missspecification. In this situation, however, it is not possible to identify from the observed yield data alone which set of trait values is more appropriate. By extension, the same applies to the sets of marker effects of which the component trait values are linear functions. This is referred to as likelihood nonidentifiability (short ‘nonidentifiability’) and is a known problem in biological modeling with hidden variables [101]. When analyses are conducted under the Bayesian statistical paradigm, nonidentifiability does not necessarily preclude inference and estimation, because informative prior distributions can identify the parameters nonetheless [102, 103]. This is another argument in favor of using informative prior distributions. Nonidentifiability also does not preclude prediction, because the posterior predictive distribution is a function that is identified even if the parameters are not [104]. If prediction is the sole purpose, the nonidentified parameters can be viewed as nuisance variables [101], that are averaged over in the posterior predictive distribution. In fact, Gianola [104] argues that in a predictive setting, parameters are merely ‘tools enabling one to go from past to future observations’. However, nonidentifiability can be associated with computational problems [102] and is of course an issue if the latent variables are of interest themselves. A special case of nonidentifiability occurs when the parameters are not identifiable for the estimation data set at hand, out of sheer coincidence [101]. However, when applied to new observations, i.e., for prediction, the parameters might be identifiable, with one set of parameters being more appropriate than the others. If this is the case, nonidentifiability might lead to a reduction in prediction accuracy.

In addition to using informative prior distributions, increasing the informativeness of the data with respect to the parameters is a direct way to improve their identifiability. In our case, this can be achieved by using additional response variables next to final grain yield. One possible choice is to use grain yield measurements from multiple environments, because several sets of component trait values might generate the same grain yield in one environment, but not in the others. Other possible choices are intermediate traits generated by the CGM, such as early biomass development or leaf area index, which can be measured non-destructively and with high-throughput [105–107].

Actually measuring the underlying physiological traits obviates the need to treat them as latent, hidden variables. This would obviously guarantee identifiability. As mentioned before, it might be possible to measure at least some of the traits. If these are key traits in the development of grain yield, observing them would identify the unobserved traits, too. One way of exploiting the information from observed physiological traits in CGM-WGP is to treat them as constants in the estimation procedure. In this framework, physiological trait values of new genotypes have to be predicted from conventional QTL or WGP models, as described above. However, CGM-WGP could also be extended to estimate marker effects for observed and hidden physiological traits simultaneously.

#### Other applications

The idea of incorporating biological insights into WGP models is not limited to CGMs. Plant metabolites are chemical compounds produced as intermediate or end products of biochemical pathways. They are seen as potential bridges between genotypes and phenotypes of plants [108] and are therefore of particular interest in plant breeding [109]. Metabolic networks model the interrelationships between genes, intermediate metabolites and end products through biochemistry pathways [110]. Elaborate metabolic network models are available today that allow studying and simulating complex biochemical processes related to crop properties, such as flowering time, seed growth, nitrogen use efficiency and biomass composition [97, 111–113]. Liepe et al. [49] demonstrated how ABC can be used for parameter estimation with metabolic and other biochemical networks. Using the principles outlined here for CGM-WGP, metabolic networks might add valuable biological information for the purpose of WGP, too.

Despite ever increasing sample sizes and marker densities, most of the genetic variance of complex traits remains unaccounted for in genome-wide association studies [114]. Marjoram et al. [51] argued that signal detection power could be increased by augmenting the purely statistical association models used thus far with biological knowledge. They demonstrated their approach by using ABC for incorporating gene regulatory networks into their analysis. Here we showed that the same principle can be applied to WGP by using ABC for integrating a CGM in the estimation of whole genome marker effects. Yield is a product of plant genetics and physiology, the environment and crop management and integrating information pertaining to these components will ultimately enable us to better predict it [115]. While this study is only a first step and many questions remain, we conclude that CGM-WGP presents a promising novel path forward towards a new class of WGP models that integrate genomics, quantitative genetics, and systems biology and thereby increase prediction accuracy in settings that have proved challenging for plant breeding and applied genetics.

## Supporting Information

S1 Table Accuracy of grain yield predictions of test DH lines with increased error in prior parameters. (PDF)

S1 Dataset SNP genotypes and trait phenotypes of synthetic maize doubled haploid lines. The data set is a representative example of the 50 synthetic data sets used in the study.
(CSV)

S1 Fig Simulated development of total and senescent leaf area. The early, intermediate and late maturing genotypes had a total leaf number (TLN) of 6, 14.5 and 23, respectively. The values for the other three traits were 750 for AM, 1.6 for SRE and 1150 for MTU and in common for all genotypes. The full and dotted vertical lines indicate the end of the 2012 and 2013 growing season, respectively.
(PDF)

S2 Fig CGM-WGP vs. GBLUP prediction accuracy in 50 synthetic data sets. (PDF)

S3 Fig Relationship between physiological traits and total grain yield. Data shown are a random sample of 1000 genotypes from a representative example replication.
(PDF)

S4 Fig Distribution of simulated grain yield in 2012 and 2013 environments. The grey lines indicate the performance of specific genotypes in both environments. Data shown is from a representative example replication.
(PDF)
