# Supplementary material for: Integrating Crop Growth Models with Whole Genome Prediction through Approximate Bayesian Computation
Source: PLoS One. 2015 Jun 29;10(6):e0130855. doi: 10.1371/journal.pone.0130855 (PMC4488317; doi:10.1371/journal.pone.0130855)
Supplement: S1 Table — (PDF) [file pone.0130855.s001.pdf]

S1 Table. Accuracy of CGM-WGP grain yield predictions of test DH lines with increasing bias in prior hyperparameters.

Estimation Env.	Prediction Env.	Prior Bias		
		0%	10%	25%
2012	2012	0.78	0.77	0.73
	2013	0.49	0.48	0.38
2013	2012	0.44	0.42	0.29
	2013	0.75	0.75	0.72

A prior bias of 10% means that the difference between the hyperparameters of the prior distributions and the true population parameters are 10% of the latter, on average. Results for prior bias of 10% are repeated from Table 2 in the main manuscript, for ease of reference. Results are average over 50 replications.
