# Supplementary material for: Integrating Crop Growth Models with Whole Genome Prediction through Approximate Bayesian Computation
Source: PLoS One. 2015 Jun 29;10(6):e0130855. doi: 10.1371/journal.pone.0130855 (PMC4488317; doi:10.1371/journal.pone.0130855)
Supplement: S1 Fig — The early, intermediate and late maturing genotypes had a total leaf number (TLN) of 6, 14.5 and 23, respectively. The values for the other three traits were 750 for AM, 1.6 for SRE and 1150 for MTU and in common for all genotypes. The full and dotted vertical lines indicate the end of the 2012 and 2013 growing season, respectively. (PDF) [file pone.0130855.s003.pdf]

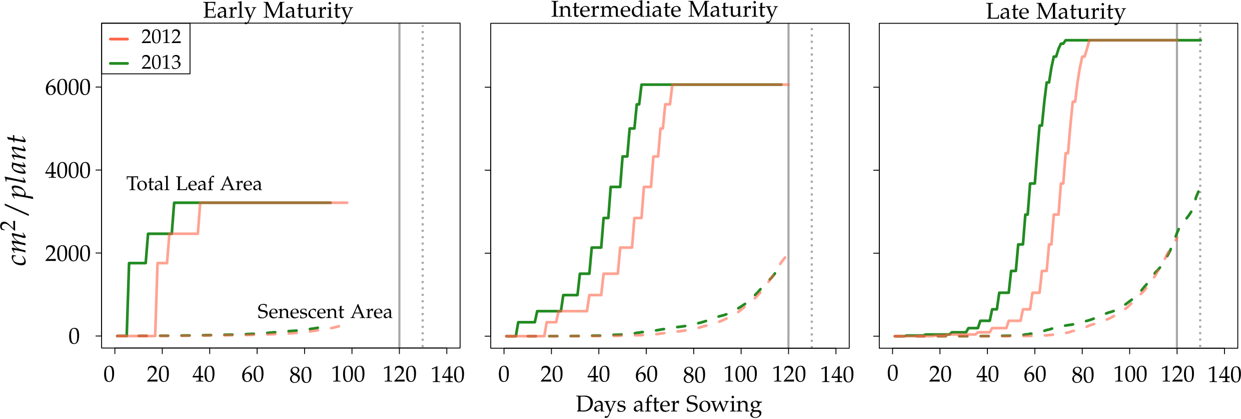

S1 Fig. Simulated development of total and senescent leaf area. The early, intermediate and late maturing genotypes had a total leaf number (TLN) of 6, 14.5 and 23, respectively. The values for the other three traits were 750 for AM, 1.6 for SRE and 1150 for MTU and in common for all genotypes. The full and dotted vertical lines indicate the end of the 2012 and 2013 growing season, respectively.
