# Supplementary figures and images for: Integrating Crop Growth Models with Whole Genome Prediction through Approximate Bayesian Computation
Source: PLoS One. 2015 Jun 29;10(6):e0130855. doi: 10.1371/journal.pone.0130855 (PMC4488317; doi:10.1371/journal.pone.0130855)

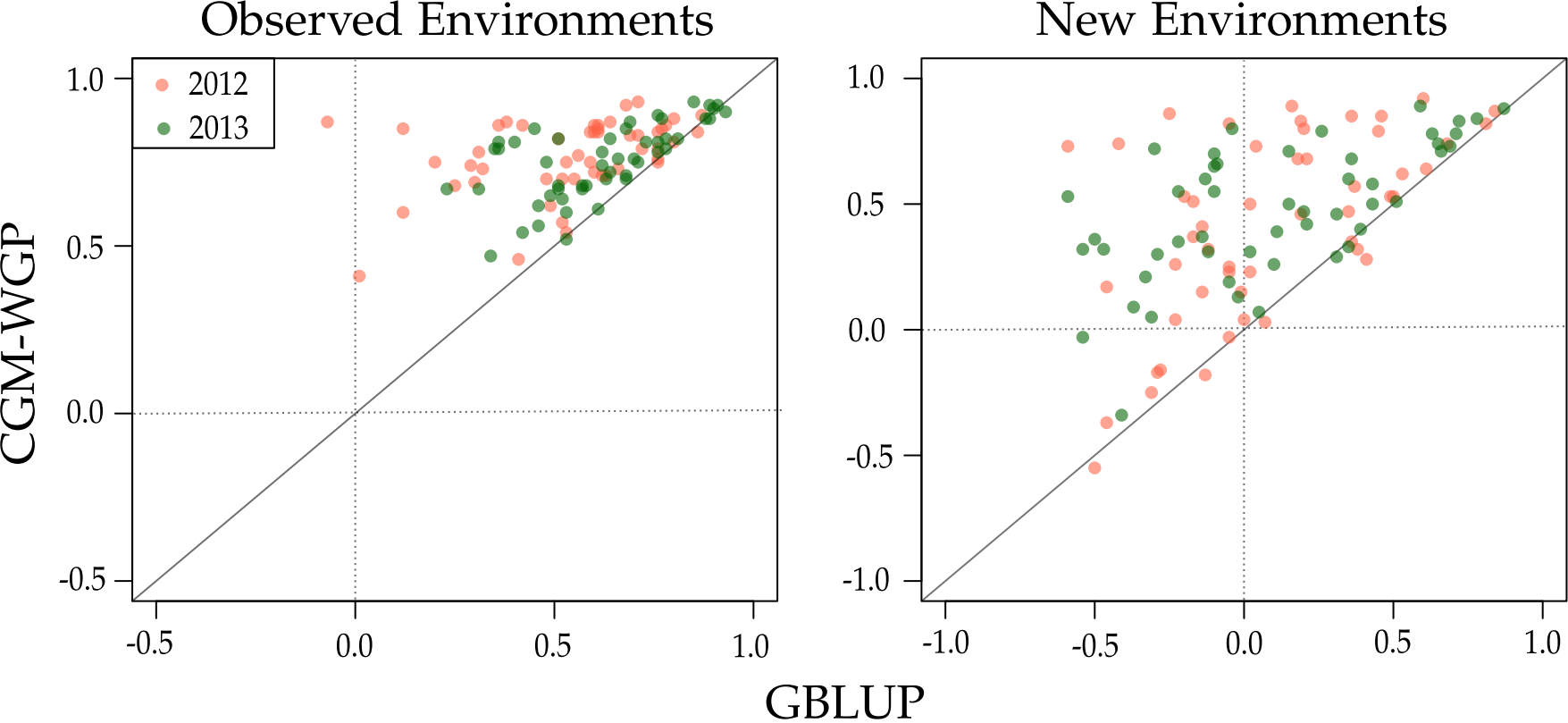

S2 Fig. CGM-WGP vs. GBLUP prediction accuracy in 50 synthetic data sets.

Supplement: S2 Fig — (PDF) [file pone.0130855.s004.pdf]
