# Supplementary material for: Integrating Crop Growth Models with Whole Genome Prediction through Approximate Bayesian Computation
Source: PLoS One. 2015 Jun 29;10(6):e0130855. doi: 10.1371/journal.pone.0130855 (PMC4488317; doi:10.1371/journal.pone.0130855)
Supplement: S3 Fig — Data shown are a random sample of 1000 genotypes from a representative example replication. (PDF) [file pone.0130855.s005.pdf]

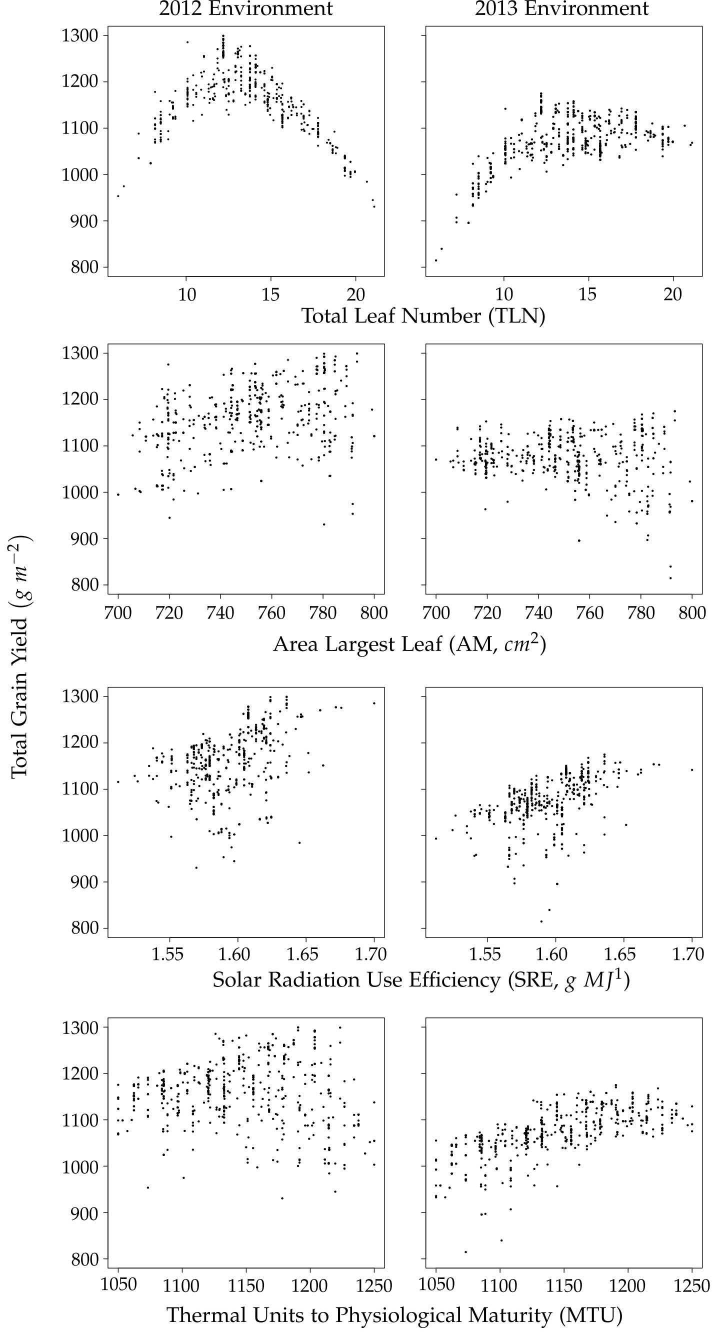

S3 Fig. Relationship between physiological traits and total grain yield. Data shown are a random sample of 1000 genotypes from a representative example replication.
