# Supplementary material for: Integrating Crop Growth Models with Whole Genome Prediction through Approximate Bayesian Computation
Source: PLoS One. 2015 Jun 29;10(6):e0130855. doi: 10.1371/journal.pone.0130855 (PMC4488317; doi:10.1371/journal.pone.0130855)
Supplement: S4 Fig — The grey lines indicate the performance of specific genotypes in both environments. Data shown is from a representative example replication. (PDF) [file pone.0130855.s006.pdf]

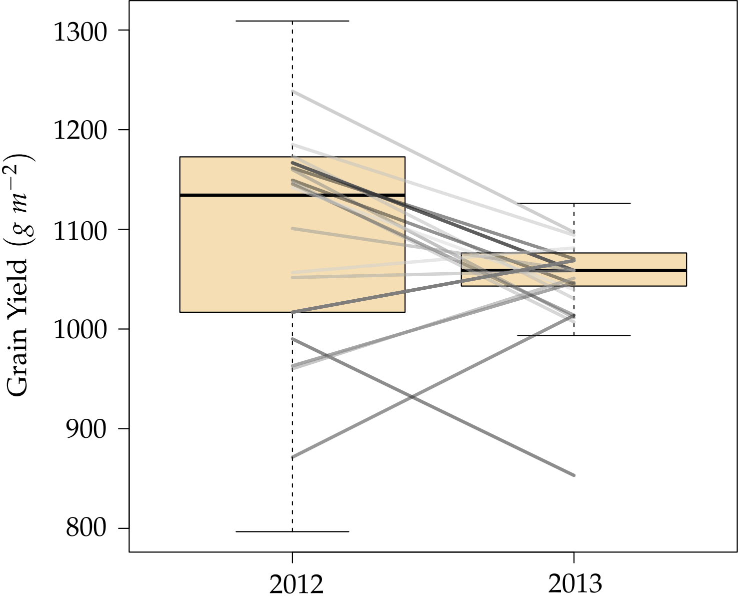

S4 Fig. Distribution of simulated grain yield in 2012 and 2013 environments. The grey lines indicate the performance of specific genotypes in both environments. Data shown is from a representative example replication.
